# Unplanned Pregnancy in women with mental disorder

**DOI:** 10.1192/j.eurpsy.2022.844

**Published:** 2022-09-01

**Authors:** A. Roca Lecumberri, E. Gelabert, A. Torres Giménez, E. Solé, S. Andrés-Perpiñá, E. Roda, C. Lopez, C. Naranjo, L. Garcia-Esteve

**Affiliations:** 1 Perinatal Mental Health Unit. Hospital Clínic de Barcelona, Insititut Clínic De Neurociències, Barcelona, Spain; 2 Universitat Autonoma de Barcelona, Clínical And Health Psychologiy Department, Barcelona, Spain

**Keywords:** unplanned pregnancy, risk factors, self-harm, perinatal mental health

## Abstract

**Introduction:**

Few studies have investigated the level of planning of pregnancy among women with mental disorder and associated risk factors.

**Objectives:**

The purpose of this study was to determine the associated factors to UP and psychopathological consequences.

**Methods:**

A cross sectional study was conducted at the Perinatal Mental Health Unit of the Hospital Clínic in Barcelona. The total sample comprised 675 consecutive pregnant women with diagnosis of mental disorder (DSM-IV criteria), seen between January 2006 and December 2018. Clinical, psychometric and socio-demographic variables were collected at the first visit. Pregnancy planning was assessed by a question “Was this pregnancy planned?” with three possible answers: 1) Yes, it was planned and has been well received; 2) No, it was not planned but it has been well received; and 3) No, it was an accident. Response 1 was coded as “planned pregnancy” and responses 2 and 3 as “Unplanned Pregnancy”.

**Results:**

38.4% of the sample had an UP. Younger age, lower levels of education, Latin-American population, multiparity, financials problems and poor relationship with the partner were associated with UP in women with mental disorder. The mean EPDS and STAI scores and the presence of self-harming thoughts were significantly higher in women with UP.

**Conclusions:**

UP was associated with more depressive and anxious symptoms and more self-harming thoughts. It is necessary to promote reproductive health care for women with mental disorders and to take into account their reproductive life plan, especially in those with risk factors described.

**Disclosure:**

No significant relationships.

